# Functional alpha7 nicotinic receptors are expressed on immature granule cells of the postnatal dentate gyrus

**DOI:** 10.1016/j.brainres.2014.12.041

**Published:** 2015-03-19

**Authors:** Danielle John, Irina Shelukhina, Yuchio Yanagawa, Jim Deuchars, Zaineb Henderson

**Affiliations:** a School of Biomedical Sciences, Faculty of Biological Sciences, University of Leeds, Leeds LS2 9JT, UK; b Department of Molecular Basis of Neurosignaling, Laboratory of Molecular Toxinology, Shemyakin and Ovchinnikov Institute of Bioorganic Chemistry, Russian Academy of Sciences, Moscow V-437, Russia; c Department of Genetic and Behavioural Neuroscience, Gunma University Graduate School of Medicine, Maebashi 371-8511, Japan; d Japan Science and Technology Agency, CREST, Sanbancho, Chiyoda-ku, Tokyo 102-0075, Japan

**Keywords:** α7^⁎^nAChR, alpha7 subunit-containing nicotinic receptor, α-btx, alpha bungarotoxin, BSA, bovine serum albumin, D-AP5, D-(−)-2-amino-5-phosphonopentanoic acid, DG, dentate gyrus, DhβE, dihydro-ß-erythrodine, GAD67, glutamate decarboxylase 67, GFP, green fluorescent protein, NBQX, 2,3,-dioxo-6-nitro-1,2,3,4-tetrahydrobenzo[f]quinoxaline-7-sulphonamide, PNU120596, N-(5-chloro-2,4-dimethoxyphenyl)-N′-(5-methyl-3-isoxazo lyl)-urea, TBA, tris-buffered ACSF, Dentate gyrus, Neurogenesis, Alpha 7 nicotinic acetylcholine receptors

## Abstract

Neurogenesis occurs throughout life in the subgranular zone of the dentate gyrus, and postnatal-born granule cells migrate into the granule cell layer and extend axons to their target areas. The α7^⁎^nicotinic receptor has been implicated in neuronal maturation during development of the brain and is abundant in interneurons of the hippocampal formation of the adult brain. Signalling through these same receptors is believed also to promote maturation and integration of adult-born granule cells in the hippocampal formation. We therefore aimed to determine whether functional α7^⁎^nicotinic receptors are expressed in developing granule cells of the postnatal dentate gyrus. For these experiments we used 2–3 week-old Wistar rats, and 2–9 week old transgenic mice in which GABAergic interneurons were marked by expression of green fluorescent protein. Immunohistochemistry indicated the presence of α7^⁎^nicotinic receptor subunits around granule cells close around the subgranular zone which correlated with the distribution of developmental markers for immature granule cells. Whole-cell patch clamp recording showed that a proportion of granule cells responded to puffed ACh in the presence of atropine, and that these cells possessed electrophysiological properties found in immature granule cells. The nicotinic responses were potentiated by an allosteric α7^⁎^nicotinic receptor modulator, which were blocked by a specific α7^⁎^nicotinic receptor antagonist and were not affected by ionotropic glutamate or GABA receptor antagonists. These results suggest the presence of functional somato-dendritic α7^⁎^nicotinic receptors on immature granule cells of the postnatal dentate gyrus, consistent with studies implicating α7^⁎^nicotinic receptors in dendritic maturation of dentate gyrus neurons in adult brain.

## Highlights

• α7^⁎^nAChRs immunofluorescently detected on immature but not mature granule cells.
• Functional nicotinic receptors found on immature granule cells.
• Nicotinic receptors of immature granule cells are somato-dendritic α7^⁎^nAChRs.
• Mature granule cells no longer possess functional nicotinic receptors.

## 1 Introduction

The dentate gyrus (DG) of the hippocampal formation, a region important for spatial and episodic memory (Lisman, 1999; Burgess et al., 2002), is a well-established site of continual neurogenesis in the mammalian brain (Altman, 1962; Altman and Das, 1965; Kaplan and Hinds, 1977; Seki and Arai, 1995; Gage, 2000; Cameron and McKay, 2001), where the processes of ontogenetic developmental neurogenesis and adult neurogenesis are considered to overlap (Amrein et al., 2011). The DG is made up of a molecular layer, granule cell layer, subgranular zone and the hilus. The molecular layer consists mainly of the dendrites of the principal neurons of the DG, i.e. the granule cells, and these dendrites receive extensive glutamatergic input from the entorhinal cortex and from mossy cells in the hilus. The granule cells themselves, densely packed into the granule cell layer, target the principal neurons in CA3 of the hippocampus and possess collaterals that synapse onto mossy cells and local GABAergic interneurons (Amaral and Dent, 1981). The GABAergic interneurons of the DG are located in the subgranular zone, hilus and molecular layer and their terminals are concentrated in the granule cell and molecular layer of the DG (Halasy and Somogyi, 1993; Houser, 2007).

During the normal development of the DG the granule cells are born in the ventricular germinal layer and in the subgranular zone, and in the adult brain these cells occupy the outer two thirds of the granule cell layer (Dayer et al., 2003). Neurogenesis continues to occur throughout life in the subgranular zone, and in the postnatal brain the newly formed neurons accumulate in the inner third of the granular layer where they differentiate and become fully integrated into the adult circuitry (Gould et al., 1999; Hastings and Gould, 1999; Wang et al., 2000; van Praag et al., 2002; Schmidt-Hieber et al., 2004; Doetsch and Hen, 2005).

Whilst much is understood about the factors that influence neurogenesis in the postnatal DG (Hagg, 2005; Zhao et al., 2008b), less is known about how the newly-generated granule cells mature and integrate into the adult circuitry of the brain. The α7 subunit-containing nicotinic receptor (α7^⁎^nAChR) is known to support neuroplasticity (Broide and Leslie, 1999; Mansvelder and McGehee, 2000; Ji et al., 2001; Kang and Vaucher, 2009) and neurite outgrowth during development (Lipton and Kater, 1989; Role and Berg, 1996; Lauder and Schambra, 1999). The receptor also plays an important role in learning, memory and attention (Dani and Bertrand, 2007) and has been shown to be required for the maturation and synaptic integration of adult-born neurons in the DG (Campbell et al., 2010).

The most common nAChR subtypes expressed in the hippocampal formation are those based on α7 and α4β2 subunits (Deneris et al., 1988; Wada et al., 1989; 1990; Seguela et al., 1993; Dominguez del et al., 1994). They are located postsynaptically on GABAergic interneurons (Alkondon et al., 1998; Frazier et al., 1998a,b, 2003) and presynaptically on GABAergic and glutamatergic axonal terminals (Colquhoun and Patrick, 1997). Localization of α7 nAChR and β2 nAChR subunits has been observed in granule cells in the DG using receptor binding and immunofluorescence respectively (Kaneko et al., 2006), but direct electrophysiological evidence for functional α7^⁎^nAChRs has been missing (Frazier et al., 2003). We therefore aimed to provide evidence for functional α7^⁎^nAChRs on granule cells in the postnatal DG and to ascertain if these receptors are expressed only by immature granule cells in the dentate gyrus of Wistar rats and in GAD67-GFP mice.

## 2 Results

### 2.1 α7(8-25) nAChR antibody characterisation

**Fig. 1:**
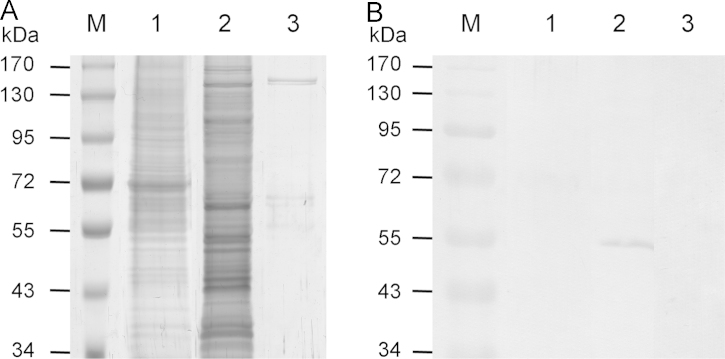
Polyclonal α7(8-25) nAChR antibody specificity characterisation by immunodetection of recombinant α7 nAChR affinity purified with α-cobratoxin-sepharose. (A) SDS-PAGE of fractions of GH4C1 cells stably expressed human α7 nAChR (silver stain). (A1) cell lysate, (A2) α-CTX affinity purified proteins; (A3) proteins non-specifically bound by CH Sepharose 4B. (B) Western blot analysis of α7 nAChR immunodetection capability for (B1) GH4C1 cell lysate, (B2) α-CTX affinity purified proteins and (B3) proteins non-specifically bound by CH Sepharose 4B. Abbrevs. M; prestained protein ladder.

The first steps of characterisation of the rabbit α7(8-25) antibody had previously been undertaken, including a series of ELISA of α7(8-25) peptide, a recombinant extracellular domain of α7 nAChR subunit and *Torpedo californica* membranes as a negative control (Shelukhina et al., 2006). In the presented work, specificity of α7(8-25) antibody to the α7 extracellular domain was confirmed by Western blot analysis (data not shown). To test immunoreactivity of the antibody for the full-length α7 subunit an approach combining α-cobratoxin affinity purification and Western blot analysis of α7 nAChR was carried out as a unique reliable knockout-proof method for immunolabelling of the receptor (Moser et al., 2007; Orr-Urtreger et al., 1997). α7(8-25) antibody did not show any unspecific labelling of unpurified original sample (Fig. 1A1 and B1) and stained a single protein band of expected molecular weight of α7 nAChR subunit (55 kDa) after its affinity purification from transfected GH_4_C_1_ cells (Fig. 1A2 and B2). Due to the previously revealed unspecific immunoreaction of commercially available antibodies (Moser et al., 2007), they were not used in this study.

Negative controls such as preincubation of the α7(8-25) antibody with excess of corresponding peptide and substitution of normal rabbit serum immunoglobulins for primary antibody eliminated any positive staining in Western blot analysis (data not shown).

### 2.2 *α*7 nAChR immunofluorescence is concentrated in the inner third of the granule cell layer of the dentate gyrus

**Fig. 2:**
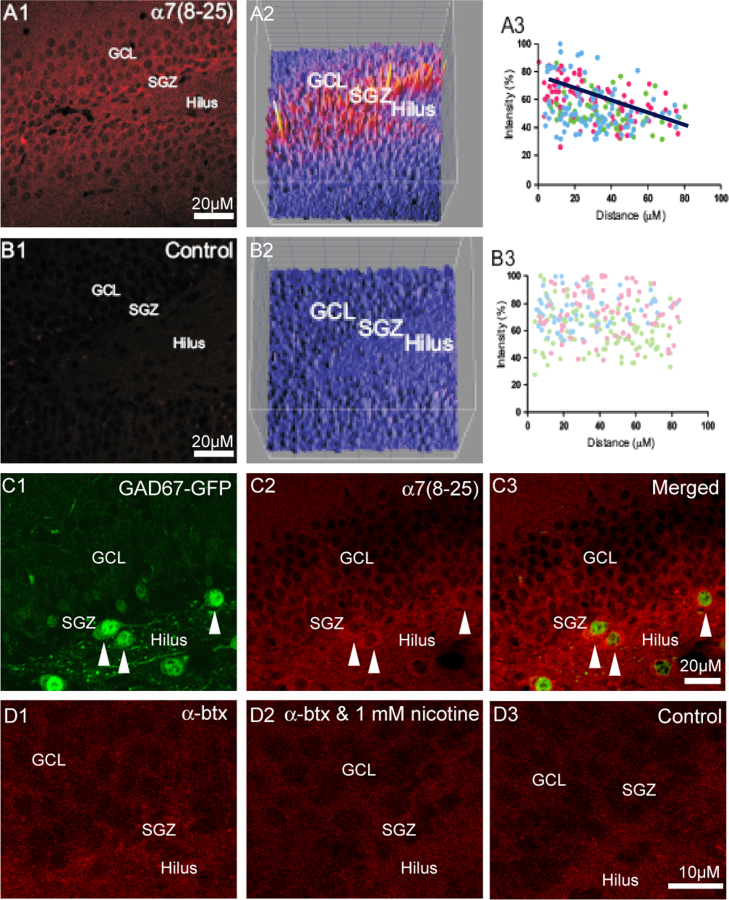
α7 nAChR subunit immunofluorescence is concentrated in the inner third of the granule cell layer of the dentate gyrus and on GABAergic interneurons. (A1) α7 nAChR subunit immunofluorescence in the rat DG. (A2) Intensity peak plot of the sample shown in (A1). (A3) Correlation between fluorescence intensity of α7nAChR subunit labelling and distance of granule cells away from the subgranular zone. (B1) Control for (A1) involving omission of the α7 nAChR antibody. (B2) Intensity peak plot of the sample shown in (B1). (B3) Correlation between fluorescence intensity and distance of granule cells away from the subgranular zone. (C) α7 nAChR subunit immunofluorescence (red) in DG of GAD67-GFP mouse in which GABAergic cells (arrowed) are labelled with GFP (green). (D1) α-bungarotoxin labelling in the rat DG. (D2) Lack of α-bungarotoxin labelling in the rat DG after co-incubation with 1 mM nicotine. (D3) Rat DG incubated with vehicle alone. Abbrevs: GCL, granule cell layer; SGZ, subgranular zone. Minor adjustments to contrast, brightness and colour balance have been made.

In rat (*n*=12) there was a higher intensity of immunofluorescence for the α7 nAChR subunit in the inner third of the granule cell layer and subgranular zone than in the outer two thirds of the granule cell layer of the DG (Fig. 2A1, A2, B1, and B2). When quantified, there was a significantly inverse correlation in the granule cell layer between the intensity of the labelling for the α7 nAChR subunit and distance from the subgranular zone (Fig. 2A3. Pearson׳s correlation test; *P*<0.005). When the primary antibody was omitted, there was no significant fluorescence and as such, no correlation (Fig. 2B3).

GABAergic interneurons in the subgranular zone of the DG, identified by GFP fluorescence in GAD67-GFP mice, possessed labelling for the α7 nAChR subunit (Fig. 2C), as expected as previous studies have indicated that GABAergic interneurons of the hippocampal formation express functional α7^⁎^nAChRs (Frazier et al., 1998a,b; Son and Winzer-Serhan, 2008). As with the data shown for rats, GAD67-GFP mice also displayed a higher intensity of immunofluorescence for the α7 nAChR subunit in the inner third of the granule cell layer and subgranular zone than in the outer two thirds of the granule cell layer of the DG (Fig. 2C). Faint label for GFP, readily distinguishable from the intense GFP label of the interneurons, was observed in a subpopulation of granule cells, as has been reported previously (Tamamaki et al., 2003). This may be a remnant of the dual GABAergic-glutamatergic nature of granule cell cells during early development (Maqueda et al., 2003).

As a further control for the positive α7nAChR subunit immunofluorescence in the DG, α-bungarotoxin (α-btx) receptor binding was carried out (rats, P21, *n*=4). α-btx identifies α7^⁎^nAChR binding sites in the CNS, and fluorescent labelling for these sites was concentrated in the subgranular zone of the granule cell layer and in the hilus (Fig. 2D1). α-btx binding was absent when 1 mM nicotine (a competitive antagonist of α7^⁎^nAChRs at this concentration) was included (Fig. 2D2), or when α-btx was omitted from the protocol (Fig. 2D3). α-btx is known to label the muscle-type nAChRs as well as neuronal α7^⁎^nAChRs, and intense α-btx labelling was seen at the neuromuscular junction in tongue sections as expected according to previously published literature (Shelukhina et al., 2009; data not shown).

### 2.3 Distribution of α7 nAChR subunit immunofluorescence coincides with that of markers of immature granule cell neurons

**Fig. 3:**
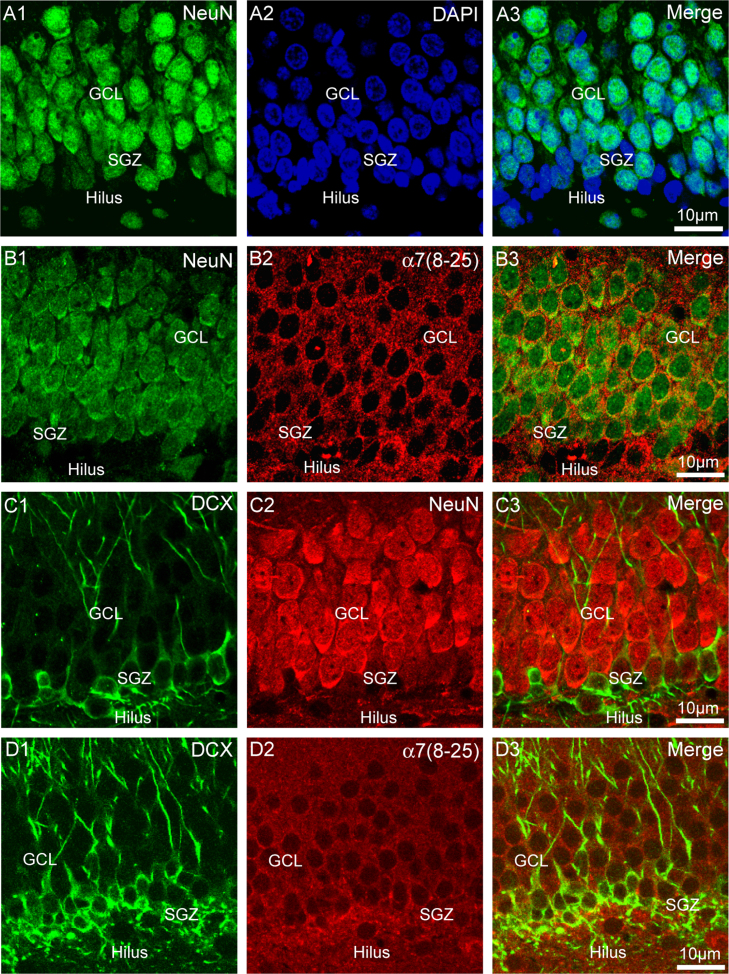
Correspondence of α7 nAChR subunit immunofluorescence with markers of immature granule cell neurons. (A) Differential intensity of labelling for NeuN (green), a marker for mature neurons, compared to DAPI (blue) between the inner and outer granule cell layer of rat DG. (B) Double label for NeuN (green) and the α7 nAChR subunit (red) in rat DG. (C) Double label for doublecortin (DCX, green) and NeuN (red) in rat DG. (D) Double label for DCX (green) and α7 nAChR subunit (red) in rat DG. Abbrevs: GCL, granule cell layer; SGZ, subgranular zone. Minor adjustments to contrast, brightness and colour balance have been made.

In rats (*n*=5) and GAD67-GFP mice (*n*=5, not illustrated), the nuclei of granule cells in the DG in the outer two thirds of the DG stained more intensely for NeuN, a nuclear marker for mature neurons (Kim et al., 2009), than the inner layer of the DG (Fig. 3A), as has been observed previously in both rats and mice (Mullen et al., 1992; Pleasure et al., 2000; Brandt et al., 2003; von Bohlen und Halbach, 2007; Snyder et al., 2009). This was reflected by the ratio of intensity of labelling of NeuN to DAPI in nuclei which was lower in the inner granule cell layer (0.6, rats, *N*=3, *n*=102; 0.6, GAD67-GFP mice, *N*=3, *n*=88) than in the outer granule cell layer (1.2, rats, *N*=3, *n*=146; 1.1, GAD67-GFP mice, *N*=3, *n*=258). In both rats and mice, the difference in the ratio of intensity of labelling was found to be statistically significant (*P*<0.001, Mann−Whitney rank sum test). In rats (*n*=3) and GAD67-GFP mice (*n*=3, not illustrated), immunostaining for the α7 nAChR subunit was stronger in the part of the granule cell layer with weak or no label for NeuN (Fig. 3B).

In rats (*n*=4) and GAD67-GFP mice (*n*=4, not illustrated), the cytoplasm of somata and dendrites of granule cells in the inner third of the DG and in the subgranular zone showed intense label for doublecortin, a marker for immature granule cells (Francis et al., 1999; Gleeson et al., 1999; Brown et al., 2003; Friocourt et al., 2003; Moores et al., 2004; Koizumi et al., 2006). The outer two thirds of the DG, where intense nuclear label for NeuN is observed, were bereft of doublecortin except in the dendrites of the underlying cells (Fig. 3C) as has been observed previously in the DG (Nacher et al., 2001). A higher level of staining for α7 nAChR subunit immunofluorescence in the DG overlapped the region with doublecortin-labelled somata; i.e. the subgranular zone and inner third of the granule cell layer on granule cells (Fig. 3D).

### 2.4 Immature but not mature granule cells possess functional α7^⁎^nAChRs

**Fig. 4:**
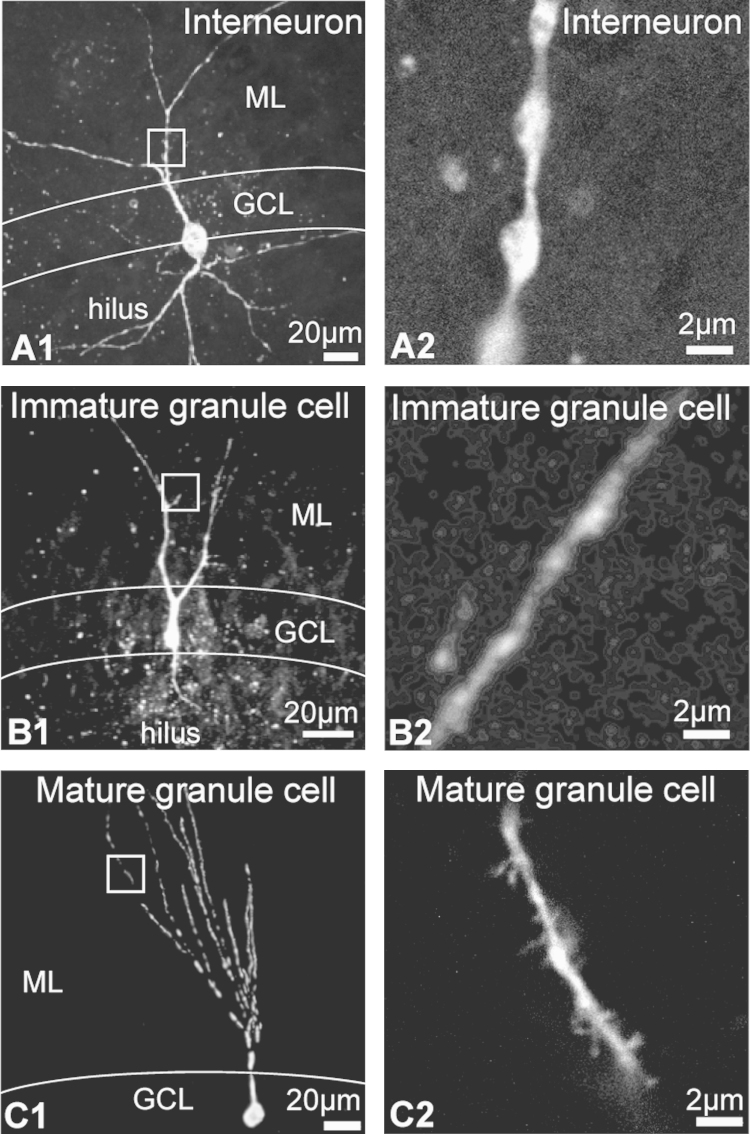
Example nAChR responses of identified cell types in the DG. Example flattened z-stack of biocytin fills from an interneuron (A1), immature granule cell (B1) and mature granule cell (C1), with a corresponding single high-resolution image from each cell type to highlight the processes (A2, B2, C2).

Electrophysiological recordings were made from neurons in live slices from both rats and GAD67-GFP mice. Neurons were identified as interneurons, immature granule cells or mature granule cells based on a number of criteria in the live slice and during post hoc analysis. The criterion for neuronal identification during recording was based on known position and appearance of the neuron in the DG (Ming and Song, 2011), and additionally on GFP content in the GAD67GFP mouse (Tamamaki et al., 2003). Interneurons are relatively large and found in the hilus, subgranular zone and molecular layer. Granule cells are relatively small, circular in shape and are confined to the granule cell layer, and previous work has indicated that postnatal-born immature granule neurons are located only in the inner third of the granule cell layer (Crespo et al., 1986). In the GAD67GFP mouse, GABAergic interneurons were identified as those that possess high levels of GFP fluorescence. Cells were recorded from and filled with biocytin and further had their identity confirmed based on their morphology as defined by previous work (Freund and Buzsaki, 1996; Wang et al., 2000; Lledo et al., 2006; Overstreet**-**Wadiche and Westbrook, 2006; Zhao et al., 2006). For example, interneurons are known to possess both basal and apical dendrites and have spiny or beaded dendrites (Fig. 4A1 and A2). Granule cells have only apical dendrites; those of immature granule neurons are poorly branched and lack spines (Fig. 4B1 and B2) whilst the dendrites of mature granule neurons are richly branched and are covered with spines (e.g. Fig. 4C1 and C2). Taking all these criteria into account, it is possible to reliably identify all cells recorded from as immature granule cells, mature granule cells or interneurons.

**Fig. 5:**
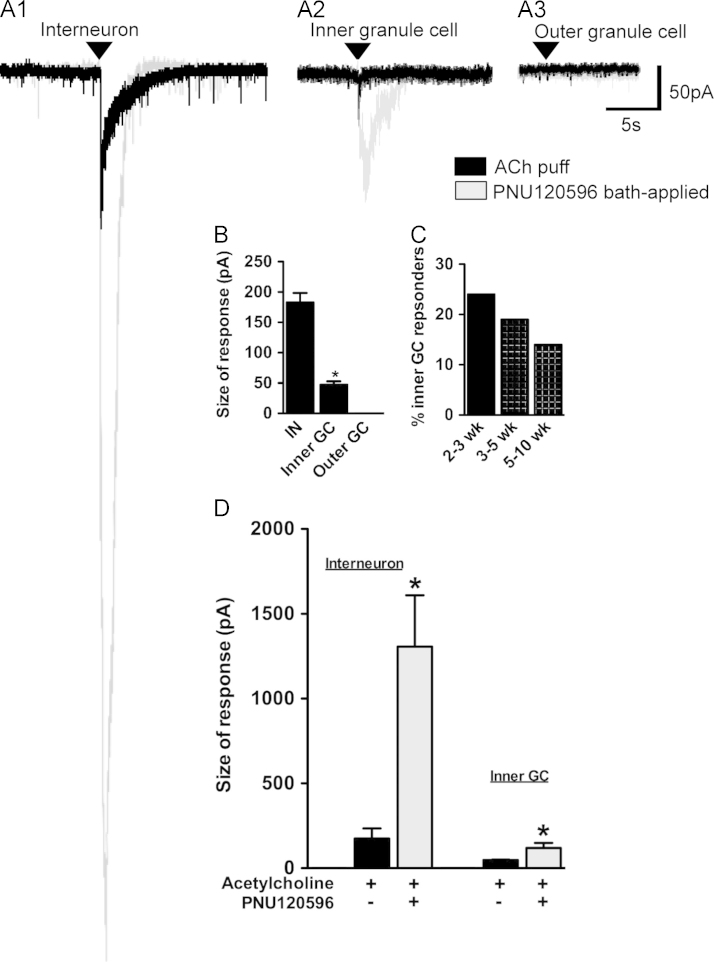
Cells of the dentate gyrus display nAChR-like responses to acetylcholine that can be potentiated by PNU120596. Example inward current responses to puff ACh (3 Mm, arrowhead) before (black trace) and after bath application of PNU120596 (grey trace, 10 μM) in (A1) interneuron, (A2) inner granule cell, and (A3) outer granule cell; same conditions of puff for each neuron; holding potential −60 mV; in the presence of 5 µM atropine). (B) Mean values for inward current responses to puff ACh for interneurons (*n*=9), inner granule cells (*n*=10), and outer granule cells (*n*=10) in GAD67-GFP mice, ^⁎^*P*<0.001. (C) Percentage of inner granule cells that respond to puff ACh versus the postnatal age (GAD67-GFP mice, 2-3 weeks postnatal, *n*=4/17; 3–5 weeks postnatal, *n*=4/21; 5–10 weeks postnatal, *n*=2/14). (D) Size of the nAChR current in response to puff of 3 mM ACh before and after 10 μM PNU120596 application in interneurons (*n*=8) and granule cells (*n*=9) (^⁎^*P*<0.05 Mann−Whitney rank sum test). All responses were recorded in the presence of 5 µM atropine and at a holding potential of −60 mV. Abbrevs: IN, interneuron; GC, granule cell.

In rats (*n*=24) and GAD67-GFP mice (*n*=55), a puff of 3 mM ACh in the presence of 5 µM atropine elicited nAChR-like responses in a proportion of interneurons (43%, *n*=22) and immature granule cells in the inner granule cell layer (17%, *n*=60) but not in mature granule cells (0%, *n*=36) in the outer two thirds of the granule cell layer (Fig. 5A1–3, black traces). The nAChR responses of the interneurons were significantly larger than those of the granule neurons (Fig. 5B). Furthermore, when the proportions of ACh-responding granule cells in the inner granule cell layer were considered according to age of the animal in the GAD67-GFP mice, there was a trend towards a decrease in the number of cells that responded to an ACh puff appeared with age, although the results were not statistically significant (Fig. 5C).

In the inner granule cell layer, nAChR responses were observed in a subset of cells that possessed action potentials, but not in cells that did not possess action potentials. Cells without action potentials in the inner granule cell layer have been shown previously to be extremely immature granule cells or glial cells (Overstreet et al., 2004), and these were not included further in the analyses. The comparison of active and passive properties of ACh-responding and non-responding neurons in the granule cell layer of rats and mice indicated that ACh-responding neurons had a significantly less hyperpolarised membrane potential, larger input resistance and a smaller and broader action potential than those of the non ACh-responding granule cells (Table 1), suggesting they are less mature than those granule cells in the inner granule cell layer that are non-responsive to ACh.

### 2.5 Nicotinic receptors of immature granule cells are of the α7^⁎^nAChR type and are somato-dendritic

**Fig. 6:**
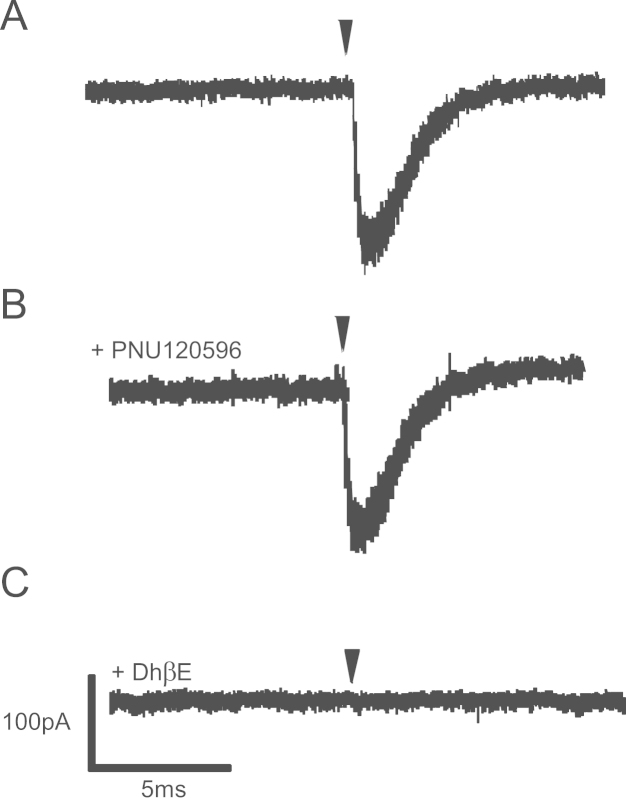
β2nAChRs are not potentiated by PNU120596. (A) Response of an interneuron to a puff of 3 mM ACh (arrowhead). (B) Response is unchanged after bath application of 10 μM PNU120596 and then puffing on 3 mM ACh (arrowhead). (C) Adding Dh*β*E to the bath abolishes the response to 3 mM ACh (arrowhead).

PNU 120596 (1–10 μM), a positive allosteric modulator of α7^⁎^nAChRs (Hurst et al., 2005), significantly potentiated the nAChR current amplitudes in interneurons (*n*=8) that had α7^⁎^nAChR-like responses (Fig. 5A), and all granule cells (*n*=9) with nAChR-like responses to ACh (Fig. 5B). These responses were blocked the α7^⁎^nAChR antagonist methyllycaconitine (4 nM, e.g. Fig. 6D) but not by the β2^⁎^nAChR antagonist DHβE (data not shown). PNU 120596 had no effect on non ACh-responding granule cells (Fig. 5C; *n*=19).

A small number of interneurons recorded from displayed cholinergic responses with β2^⁎^nAChR characteristics (*n*=3), i.e. their responses to puffed ACh was characterised by a rounded downward current with a much slower upstroke than the α7^⁎^nAChR-like response (Fig. 6A). These responses were not potentiated by 1–10 μM PNU120596 (Fig. 6B) and were blocked by 20 nM DhβE, a specific antagonist of the β2^⁎^nAChRs (Fig. 6C).

**Fig. 7:**
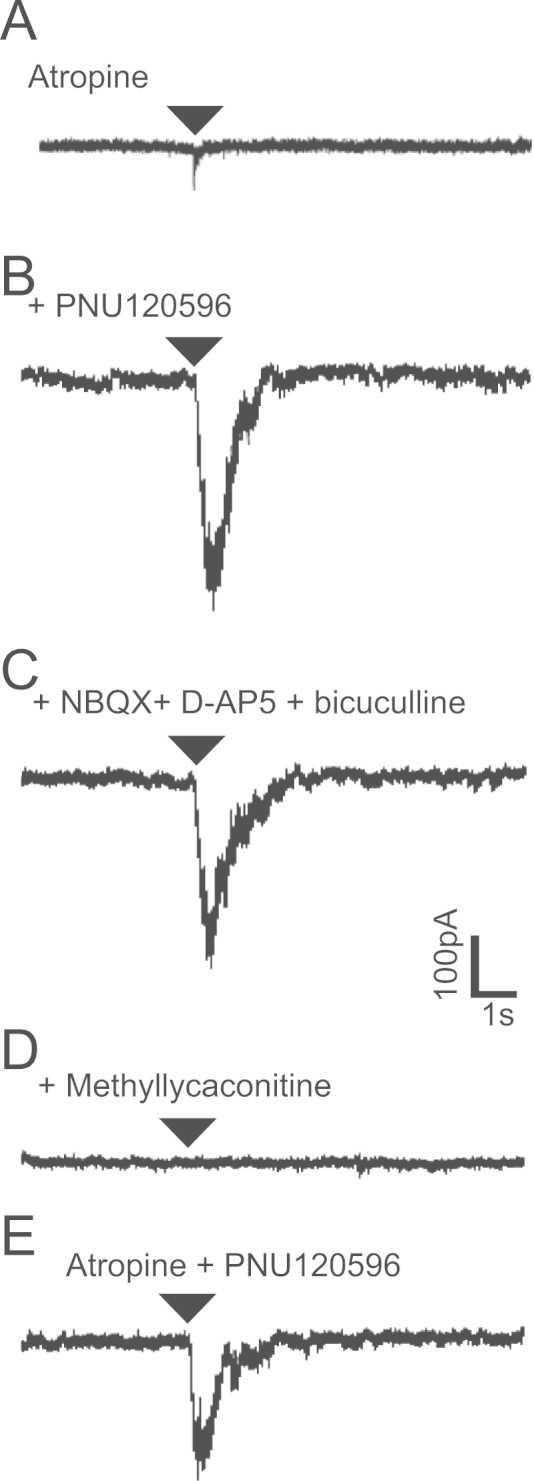
α7*nAChRs on immature dentate granule cells are somato-dendritic. Responses of a granule cell neuron to puff 3 mM ACh (A) in the presence of 5 µM atropine, (B) after addition of 10 μM PNU120596, (C) after addition 10 μM NBQX, 25 μM D-AP5 and 10 μM bicuculline, (D) after addition of 4 nM methyllycaconitine, and (E) after wash in the presence of 5 µM atropine and 10 μM PNU120596 alone.

nAChR responses in granule cells that were potentiated by PNU120596 were not blocked by the ionotropic glutamate receptor blockers 10 μM NBQX and 25 μM D-AP5, or by the ionotropic GABA receptor blocker 10 μM bicuculline (*n*=3), but were readily inhibited by the α7^⁎^nAChR antagonist methyllycaconitine (4 nM, Fig. 7; *n*=8). These results suggest that immature granule cells in the postnatal dentate gyrus possess functioning somato-dendritic α7^⁎^nAChRs.

## 3 Discussion

### 3.1 Conclusions

Using immunohistochemistry we have shown, in both rats and mice, the presence of α7 nAChR subunits around granule cells close to the germinal layer in the subgranular zone. These findings were confirmed with α-bungarotoxin labelling. This immunohistochemical labelling corresponds with the distribution of developmental markers for immature granule cells such as weak nuclear label for NeuN and presence of doublecortin. Electrophysiology using whole-cell patch clamp recording indicated that a proportion of granule cells in the inner granule cell layer with electrophysiological properties of immature neurons (such as relatively broad action potentials and high input resistance), respond to puffed ACh in the presence of atropine. These responses are nicotinic in nature and are mediated by α7^⁎^nAChRs since they are potentiated by PNU 120596 (a known positive allosteric modulator of α7^⁎^nAChRs), inhibited by methyllycaconitine but not DHβE, and not affected by ionotropic glutamate or GABA receptor antagonists. The same nAChR responses were not seen in granule cells in the outer granule cell layer that are presumed to be mature and possess mature electrophysiological properties. These results suggest the presence of functional somato-dendritic α7^⁎^nicotinic receptors on immature granule cells of the dentate gyrus.

Although concern has been raised over the specificity of certain commercially available antibodies directed against epitopes in the α7 nAChR subunit (Herber et al., 2004; Moser et al., 2007), our non-commercially produced antibody was well characterised by ELISA and Western blot analysis. Furthermore, the obtained immunohistochemical results were substantiated with α-bungarotoxin labelling, and is in agreement with previous work in rat that indicates that principal cells of the DG express α7^⁎^nAChR subunit mRNA (Adams et al., 2002; Son and Winzer-Serhan, 2008). Furthermore, our electrophysiology and receptor binding results reflected the receptor binding expression obtained with autoradiographic methods for rats of the same age as we used (Adams et al., 2002).

Previous electrophysiology studies have indicated the presence α7^⁎^nAChR responses in a proportion of CA1 principal neurons, and which were accentuated in transgenic mice that expressed a mutant form of α7^⁎^nAChR that does not desensitize as rapidly as the native receptor (Ji and Dani, 2000). DG granule cells, however, were apparently unresponsive to focal somatic or dendritic application of ACh, irrespective of whether their soma was located in the inner or outer granule cell layer (Frazier et al., 2003). A possible reason for the discrepancy is that we found the responses of the native receptors to ACh on granule cells were small and only in a proportion of granule cells in the inner layer. These responses however were accentuated by PNU 120596, a positive allosteric modulator of α7^⁎^nAChRs with little or no activity on most other nAChRs (Hurst et al., 2005; Gronlien et al., 2007; Young et al., 2008) and which acts as a cognitive enhancer in vivo (Ng et al., 2007;Timmermann et al., 2007). Our studies also indicated that the ACh-responsive cells in the granule cell layer of the DG have the active and passive membrane properties expressed by immature adult-generated granule cells (Ambrogini et al., 2004; Schmidt-Hieber et al., 2004; Esposito et al., 2005; Doetsch and Hen, 2005; Lledo et al., 2006; Overstreet**-**Wadiche and Westbrook, 2006).

In our study we used doublecortin as a developmental marker for immature granule cells. Alternative markers to doublecortin are PSA-NCAM (Wang et al., 2000; Cameron and Mckay, 2001) and TOAD-64/TUC-4/CRMP4 (Cameron and McKay, 2001; van Praag et al., 2002; Ming and Song, 2005; von Bohlen und Halbach, 2007; Taupin, 2007). Other potential markers include Tuj-1β, but this labels only very immature DG neurons, i.e. before they have action potentials, and also labels non-neuronal cells and not all the neural cells (Kempermann et al., 2004; Doetsch and Hen, 2005; Lledo et al., 2006; von Bohlen und Halbach, 2007). Calretinin and calbindin have been used as markers for immature and mature cells respectively (Kempermann et al., 2004; Lledo et al., 2006; von Bohlen und Halbach, 2007), but early trials with these markers in our hands suggested that they do not label all candidate cells.

### 3.2 Role of α7^⁎^nicotinic receptors in maturation of dentate gyrus neurons in postnatal brain

The precise function of postnatal-born granule cells is widely debated, but it is significant that these cells are preferentially recruited over older granule cells into circuits supporting spatial memory (Kee et al., 2007; Clelland et al., 2009). It is likely that postnatal-born granule cells replace older degenerating neurons in the DG circuit (Zhao et al., 2008a) and have specific properties that facilitate learning (Snyder et al., 2001; Shors et al., 2001, 2002).

α7^⁎^nAChRs would have an important function in postnatal-born DG granule cells because these receptors mediate three types of cytoplasmic calcium signals, direct calcium influx through the receptor, indirect calcium influx via the activation of voltage-gated calcium channels, and calcium-induced calcium release from the endoplasmic reticulum via ryanodine receptors or inositol (1,4,5)-triphosphate receptors (Shen and Yakel, 2009). These regulate cytoplasmic calcium levels and transcriptional events involving CaMKII/IV, ERK/MAPK and CREB (Greenberg et al., 1986; Nakayama et al., 2001; Chang and Berg, 2001; Hu et al., 2002; Dajas-Bailador et al., 2002), i.e. signalling cascades that are central to long-term plasticity in the central nervous system (Sweatt, 2001). They are also of physiological relevance to addiction, learning and memory (Bliss and Collingridge, 1993; Nestler, 2002; Malenka and Bear, 2004).

One hypothesis for the function of nAChRs is to potentiate long-term potentiation (LTP) exclusively in the immature granule cells, especially as LTP is induced more readily in immature adult-born granule cells than in mature granule cells (Wang et al., 2000; Schmidt-Hieber et al., 2004). Furthermore, enhancement of LTP by α7^⁎^nAChR stimulation has been observed in CA1 (Fujii et al., 2000; Matsuyama et al., 2000; Ji et al., 2001; Lagostena et al., 2008), ventral tegmental area (Ge and Dani, 2005) and the DG (Welsby et al., 2006, 2007, 2009); in the latter case the LTP it may be mediated by either interneurons or immature granule cells or both.

Integration of the adult-born granule cells is indicated by a recent study using α7^⁎^nAChR knock-out mice injected with BrdU: in these preparations the adult-born neurons develop with truncated, less complex dendritic arbours and display GABAergic postsynaptic currents with immature kinetics and they have a prolonged period of GABAergic depolarization characteristic of an immature state (Campbell et al., 2010). The location and temporal expression pattern combined with electrophysiological functionality, suggest that α7^⁎^nAChRs may play a role in maturation and synaptic integration of adult-born immature granule cells into the existing circuitry of the dentate gyrus.

### 3.3 α7 nAChRs as a therapeutic target for the treatment of Alzheimer׳s disease

Continuing neurogenesis throughout life has been observed not only in mice and rats but also in macaques (Gould et al., 2001) and humans (Eriksson et al., 1998). Nicotine and selective α7^⁎^nAChRs agonists have been shown to improve cognitive performance in animal models and humans (Van Kampen et al., 2004; Newhouse et al., 2004; Hajos et al., 2005) and certain types of memory (Dani and Bertrand, 2007; Kenney and Gould, 2008). The cholinergic innervation of the hippocampal formation, including the DG, arises from the medial septum (Kasa, 1986; Woolf, 1991), and as with other parts of the central nervous system, the cholinergic innervation of the hippocampal formation is generally diffuse, suggesting broad, modulatory roles for cholinergic signalling at both muscarinic receptors and nAChRs (Frotscher and Leranth, 1985; Kasa, 1986;Umbriaco et al., 1995; Descarries et al., 1997). Axo-somatic connections from the largely cholinergic medial septum have nevertheless been found to exist on adult-born DG granule cells soon after they have been generated (Ide et al.**,** 2008). Degeneration of the cholinergic terminals in the hippocampal formation gives rise to the early cognitive defects seen in Alzheimer׳s disease, but the α7^⁎^nAChRs seem to be unaffected (Auld et al., 2002). Since our studies suggest that α7^⁎^nAChRs are expressed at higher intensities on immature granule cells in the postnatal DG, one therapeutic avenue of action of specific α7^⁎^nAChRs agonists could therefore be via these cells in the DG and these could be a target for the treatment of Alzheimer׳s disease. In fact, one study even shows that galantamine, an acetylcholinesterase inhibitor used in the treatment of Alzheimer׳s disease, promotes adult DG neurogenesis via α7^⁎^nAChRs (Kita et al., 2014), making this a very promising avenue for future research.

## 4 Experimental procedure

### 4.1 Ethical approval

Tissue preparation procedures were carried out in accordance with the UK Animals (Scientific Procedures) Act 1986 and associated guidelines, and with prior approval from the local ethical committee of the University of Leeds. Every effort was made to minimize animal suffering and to reduce the number of animals used.

### 4.2 Tissue preparation

Studies were made on brains from 2 to 3-week-old male Wistar rats and from 2 to 9 week-old heterozygous male and female GAD67-GFP (Δneo) mice that had been bred at the University of Leeds. In the transgenic mice, glutamate decarboxylase 67 (GAD67), a specific marker for GABAergic neurons, is co-expressed with green fluorescence protein (GFP). The mice have been described further elsewhere (Tamamaki et al., 2003; Henderson et al., 2010). It is unclear exactly how long it takes newborn granule cells of the postnatal DG to become fully integrated within the existing circuitry, as this ranges 3 to 8 weeks after neurogenesis (Shors et al., 2001, 2002; Snyder et al., 2001; van Praag et al., 2002; Jessberger and Kempermann, 2003; Kempermann et al., 2004; Schmidt-Hieber et al., 2004; Bruel-Jungerman et al., 2005; Esposito et al., 2005; Ming and Song, 2005; Zhao et al., 2006; Kee et al., 2007; Toni et al., 2007). One study claims that the genetic composition of intrinsic factors within the precursor cell population in mice may take until postnatal day 60 to become fully established as an adult phenotype (Gilley et al., 2011), which is reflected in the age range studied here.

### 4.3 Immunofluorescence

The objective of the immunofluorescence experiments was to examine the distribution of α7^⁎^nAChR immunoreactivity in DG of rat and transgenic mouse brains in relation to various other relevant markers for developmental stages of the DG. The general procedures and controls used for the immunofluorescence were as described previously (Henderson et al., 2010). Wistar rats (*n*=17) and GAD67-GFP (Δneo mice) (*n*=9) were deeply anaesthetised with an intraperitoneal injection of urethane (12 g kg^−1^) or Sagatal (sodium pentobarbitone, 100 mg kg^−1^, Rhône Mérieux Ltd., Harlow, Essex, UK). After loss of all pedal and corneal eye reflexes the animals were perfused trans-cardially with 4% paraformaldehyde in 0.1 M phosphate buffer (pH 7.4). The brains were removed and placed in the same fixative for 1−2 h, and then in phosphate buffer overnight at 4 °C. Sections were cut at 50 μm in the coronal plane using a Leica VT1000S vibratome (Leica, Microsystems UK, Milton Keynes, UK) and washed in phosphate buffered saline (PBS, pH 7.4), the solution used for all wash procedures. Antigen retrieval for sections stained for the α7 nAChR subunit was carried out by incubation of the sections in 50% ethanol for 30 min. The sections were incubated for 1 h in 2% bovine serum albumin (BSA) and then placed in single or double antibody solutions in 2% BSA overnight at room temperature or for up to 3 nights at 4 °C. The following antibody solutions and dilutions were used: 1:500 goat anti-doublecortin (Santa Cruz Biotechnology, Heidelberg, Germany), 1:1000 mouse anti-NeuN (Chemicon, Millipore UK, Watford, UK), and 1:1500 of a rabbit antibody raised against residues 8-25 of the α7 nAChR subunit (Shelukhina et al., 2006;Tsetlin et al., 2007). Following washes, the sections were incubated for 2 h in appropriate combinations of 1:1000 donkey secondary antibody against mouse, rabbit or goat IgG, conjugated to Alexa Fluor 594 or Alexa Fluor 555 for red fluorescence, or to Alexa Fluor 488 for green fluorescence (Invitrogen Life Technologies, Paisley, UK). The sections were then washed and mounted on Polysine^®^ slides (Fisher Scientific UK Ltd., Loughborough, UK) and embedded under coverslips in Vectorshield mounting medium either with or without 4′,6-diamidino-2-phenylindole to label nuclei (DAPI; Vector Laboratories, Burlingame, CA, USA).

### 4.4 Alpha-bungarotoxin labelling

This receptor binding method was adapted from previous studies (Jones and Wonnacott, 2004; Oddo et al., 2005; Shelukhina et al., 2009). Wistar rats (P21, *n*=4) were deeply anaesthetised with an intraperitoneal injection of Sagatal (sodium pentobarbitone, 100 mg kg^−1^, Rhône Mérieux Ltd., Harlow, Essex, UK). After loss of all pedal and corneal eye reflexes the animals were perfused trans-cardially with ice-cold 5% sucrose dissolved in Tris-buffered ACSF (TBA) that had the following components in mM: Tris, 50; NaCl: 120, KCl: 5, CaCl_2_: 2.5: MgCl_2_: 1, and a pH of 7.4. Frozen, unfixed tissue from was trimmed, and 20 µm horizontal sections of the hippocampal formation and tongue were sectioned on a cryostat (Leica). The sections were thaw-mounted on Polysine^®^ slides and stored at −80 °C until required. All solutions were made in 0.1% Triton in TBA, and reactions were carried out in Coplin jars on a shaker. Selected sections were removed from the freezer and brought to room temperature, and fixed in isopropyl alcohol for 10 min. The sections were incubated in 1% BSA in TBA for 30 min and were then incubated overnight at 4 °C in either 50 nM α-bungarotoxin-biotin (Invitrogen, Paisley, UK) in 1% BSA in TBA, 50 nM α-bungarotoxin-biotin and 1 mM nicotine (or 10 µM α-cobratoxin) in 1% BSA in TBA, or in vehicle alone. The sections were washed 5 times in TBA and then in 1/1000 streptavidin-594 or 488 in 1% BSA in TBA for 1 h at room temperature.

### 4.5 Western blot analysis

This method was employed for examination of specificity of a rabbit antibody raised against a synthetic fragment 8-25 of rat α7 nAChR (Shelukhina et al., 2006). Recombinant extracellular domain of α7 nAChR subunit (Korotina et al., 2003) was used as a model antigen. For examination of the antibody immunoreactivity for the full-length α7 nAChR subunit a lysate and α-cobratoxin-affinity purified fraction of GH_4_C_1_ cells over-expressing human α7 nAChR (Tsetlin et al., 2007) were prepared. It should be noted that the human and rat α7(8-25) sequence (YKELVKNYNPLERPVAND) share 100% homology. For this purpose, GH_4_C_1_ cells (8 mg of protein) were resuspended in 10 ml of lysis buffer containing 20 mM sodium phosphate, pH 8.0, 1 mM EDTA, protease inhibitor cocktail, 1% Triton X-100 and shaken overnight at 4 °C. After centrifugation at 10,000*g* for 30 min 0.5 ml of supernatant was separated for SDS-PAGE and Western blot analysis (Fig. 1A1, B1, lysate), the rest was shaken overnight at 4 °C with 30 µl of α-cobratoxin coupled to CH Sepharose 4B (GE Healthcare, Sweden). Preparation of the activated CH Sepharose 4B and coupling procedure (5 mg toxin/ml medium) were performed according to the manufacturer׳s instruction. To control nonspecific protein sorption the lysate was incubated with 30 µl of uncoupled CH Sepharose 4B (Fig. 1A3). Both sepharoses were recovered by centrifugation at 1000*g* for 5 min and washed four times with 1 ml of the lysis buffer. Bound proteins were eluted with 40 µl of SDS/sample buffer and separated by 10% SDS-PAGE followed by transfer to an Immobilon membrane (Millipore, MA, USA). The membrane was blocked for 2 h with 5% dry milk in PBS and then incubated overnight at 4 °C with antibodies to α7(8-25) (30 µg/ml) in 0.5% dry milk and 0.1% Tween 20 in PBS. The membrane was washed and probed with a donkey-anti-rabbit IgG antibody coupled to peroxidase (Amersham Biosciences, Sweden) at a dilution of 1:1500. After wash, peroxidase activity was detected using SIGMA*FAST*^™^ 3,3′-Diaminobenzidine tablets (Sigma-Aldrich, USA). As negative controls preincubation of the primary antibody with 10-fold molar excess of α7(8-25) peptide for 3 h and substitution of normal rabbit serum immunoglobulins for the α7(8-25) antibody were performed.

### 4.6 Electrophysiology

Electrophysiological experiments were carried out to determine if DG granule cells had functional α7^⁎^nAChRs. Wistar rats (*n*=24) and GAD67-GFP mice (*n*=55) were anaesthetised by intraperitoneal injection of Sagatal (sodium pentobarbitone, 100 mg kg^−1^, Rhône Mérieux Ltd., Harlow, UK). When all pedal and corneal eye reflexes were abolished, the animals were perfused intracardially with chilled (5 °C), oxygenated artificial cerebrospinal fluid (aCSF) in which the sodium chloride had been replaced by iso-osmotic sucrose. This aCSF (305 mosmol l^−1^) contained (in mM): 225 sucrose, 3 KCl, 6 MgSO_4_, 0.5 CaCl_2_, 1.25 NaH_2_PO_4_, 24 NaHCO_3_ and 10 glucose. Slices of brain of thickness 300 μm for mice and 350 μm for rats were cut in the horizontal plane (i.e. to produce transverse hippocampal slices) at 5 °C in the sucrose aCSF using a Leica VT1000S vibratome (Leica Microsystems UK, Milton Keynes, UK).

Whole cell patch recordings were carried out as described previously (Henderson and Jones, 2005; Henderson et al., 2005). Slices were maintained for at least 1 h at room temperature in a holding chamber, just beneath the surface of aCSF bubbled with carbogen gas (95% O_2_–5% CO_2_). In the recording bath, the slices were maintained at 34 °C and submerged in oxygenated aCSF solution (flow rate 2.1 ml min^−1^). This ACSF (305 mosmol l^−1^) contained (in mM): 126 NaCl, 3 KCl, 2 MgSO_4_, 2 CaCl_2_, 1.25 NaH_2_PO_4_, 24 NaHCO_3_ and 10 glucose. Whole-cell patch recordings were made with micropipettes (resistances 4–6 MΩ) that contained (mM): 140 K gluconate; 5 KCl; 2 MgCl_2_; 10 HEPES; 0.1 EGTA; 0.025 CaCl_2_; 2 ATP-Na; 0.4 GTP-Na (pH 7.35, 280 mosmol l^−1^). Biocytin (0.5 mg) was mixed into 1 ml of patch solution just before use and filtered as used. Recordings were made using an AxoClamp 2B amplifier (Axon Instruments Inc., Union City, CA, USA) from somata visualized by infrared differential interference contrast video-microscopy (Zeiss Axioscope microscope, Hamamatsu CCD camera, Luigs and Neumann Infrapatch set-up, Ratingen, Germany). Images were captured by a frame grabber (Scion Corporation, Alrad Instruments Ltd., Newbury UK) and processed with CorelDraw X. Granule cells, located in the granule cell layer, were recorded from. GFP-positive GABAergic interneurons were identified and recorded as described previously (Henderson et al., 2005). A giga-seal resistance was obtained before acquiring the whole-cell recording mode. Recordings were analogue filtered at 1–3 kHz and digitized at 5–10 kHz with an ITC-16 ADC board (Digitimer Ltd., Welwyn Garden City, Hertfordshire, UK) and Axograph software (Axon Instruments). Electrical interference from the mains supply was suppressed with the use of a 50 Hz noise eliminator (Humbug; Digitimer Ltd.).

To correlate pharmacological responses with neuron cell type, the passive and regenerative membrane properties of each neuron were characterized in current clamp mode before the pharmacological studies were carried out, and analysed offline, as described previously (Henderson et al., 2005). In brief, membrane potential was measured on break-in, input resistance was calculated from the measurement of the average size of the voltage response to 20 pA hyperpolarising current pulses. Firing properties were determined by application of 1000 ms depolarising steps of 10–200 pA. For cells that fired a train of action potentials, the first three action potentials from the first train were measured. For cells that only fired single action potentials, the first three single action potentials were measured. nAChR responses were then characterised in voltage clamp mode at a holding potential of −60 mV, during which patch seal integrity was monitored by application of 5 mV, 50 ms hyperpolarizing voltage pulses every 60 s. ACh (3 mM in HEPES buffered aCSF) was puffed onto the cells from a pipette identical to those used for whole cell recording, with the use of a PicoPump (World Precision Instruments, Stevenage, Hertfordshire, UK), as described previously (Henderson et al., 2005). The composition of the HEPES buffered aCSF vehicle (also used as a negative control) was as follows (mM): 146 NaCl; 10 HEPES; 2.5 KCl; 2 CaCl_2_; 2 MgCl_2_; 5 glucose (pH 7.3; 310 mosmol l^−1^). All other drugs were applied via bath perfusion. ACh puff was done at every 180 s to prevent desensitization, first without atropine and then in the presence of 5 µM atropine. The puff pipette tip was placed at a distance of 20–50 μm from the neuron from which the recording was made, and the ACh was applied for 5–15 ms at a time and at a pressure of 20–25 psi.

To visualise biocytin-filled cells, the slice was removed from the recording chamber after recording and fixed overnight in 4% paraformaldehyde in 0.1 M phosphate buffer, washed several times in 0.1% phosphate buffer and left overnight in phosphate buffer at 4 °C. Slices were embedded in 10% gelatin (Porcine type A) in 0.1 M phosphate buffer at 40 °C for 30 min. After setting of the gelatin, sections were cut at 75 µm using the Leica VT1000S vibratome, washed in PBS and then incubated in 1:1000 streptavidin Texas Red (Invitrogen) for 2 h. Some slices were processed for biocytin by using a standard diaminobenzidine histochemical method as described previously (Henderson et al., 2004).

All standard reagents used for the electrophysiology experiments were obtained from VWR International (Lutterworth, Leicestershire, UK), Fisher Scientific Ltd. (Loughborough, Leicestershire, UK) or Sigma (Poole, Dorset, UK). The following drugs were obtained from Tocris Cookson Ltd. (Bristol, UK): D-(−)-2-amino-5-phosphonopentanoic acid (D-AP5), 2,3,-dioxo-6-nitro-1,2,3,4-tetrahydrobenzo[f]quinoxaline-7-sulphonamide (NBQX), N-(5-chloro-2,4-dimethoxyphenyl)-N′-(5-methyl-3-isoxazo lyl)-urea (PNU 120596) and bicuculline. Atropine, methyllycaconitine and dihydro-ß-erythrodine (DHßE) were obtained from Sigma (Poole, Dorset, UK). Stock solutions, at 10^3^ of the working concentration, were made up in water, except for NBQX which was dissolved in dimethylsulphoxide and stored in individual aliquots at −45 °C. Working solutions were prepared freshly on the day of the experiment.

### 4.7 Image acquisition and analysis

Sections with fluorescent markers were viewed, and images were taken at random through the rostro-caudal axis of the DG using a Zeiss LSM 510 Meta confocal microscope (Zeiss, Welwyn Garden City, UK) equipped with helium/neon, argon and diode 405 nm lasers. The signals emanating from the red (Alexa Fluor 594/555), green (Alexa Fluor 488) and blue ( Alexa Fluor 405) fluorescent labels were acquired via the 543 nm, 488 nm and 405 nm excitation bands of the lasers, respectively. For each field of view, three to seven confocal images were captured as a stack starting from the surface of the section, with the images taken at intervals of 3–4 µm for cell size measurements. Counts and measurements of cells were made using the Zeiss LSM Image Browser. Intensity measurements where appropriate were carried out using ImageJ freeware (1.43 N, Wayne Rasband, National Institutes of Health, USA).

### 4.8 Statistical analyses

All statistical tests were performed using SigmaStat software (SPSS Inc., California, USA). Results are expressed as mean±standard error of mean except where stated otherwise. The strength of the association between the variables was assessed using the Pearson product moment correlation test. Statistical significance for comparison between two groups was determined with Student׳s *t* test or the Mann−Whitney rank sum test. Statistical comparisons for more than two groups were made using one way analysis of variance. Measures were considered statistically significant if *P*<0.05.

## Author contributions

DJ and ZH conceived and designed the experiments. DJ, IS and ZH collected, analyzed and interpreted the data. YY provided the GAD67-GFP (Δneo) mouse line. DJ, IS, YY, JD and ZH were involved with drafting the article or revising it critically for important intellectual content. All authors approved the final version of this manuscript.

## Figures and Tables

**Table 1:** Membrane properties for ACh-responding and non-responding granules cells in mouse DG.

	**ACh-responding granule cells from the inner third of the granule cell layer**	**Non-responding granule cells from the outer third of the granule cell layer**
Resting membrane potential (mV)	−55±3 (*n*=10)	−65±3 (*n*=23)⁎
Input resistance (MΩ)	976±136 (*n*=10)	463±33 (*n*=23)⁎⁎
Action potential height (mV)	66±5 (*n*=10)	73±7 (*n*=23)⁎
Action potential width (ms)	2.2±0.1 (*n*=10)	1.9±0.2 (*n*=23)⁎
Action potential rise time (ms)	0.9±0.1 (*n*=10)	0.7±0.04 (*n*=23)⁎

Action potential height measured from voltage threshold to peak. Action potential width measured as the width at voltage threshold. Action potential rise time measured as the time from voltage threshold to peak of action potential. The ACh-responding granule cells were in the inner third of the granule cell layer and the non-ACh-responding granule cells were in the outer third of the granule cell layer.

⁎ *P*≤0.05, Mann−Whitney rank sum test.

⁎⁎ *P*≤0.01, Mann−Whitney rank sum test.
